# Regulation of neural process growth, elaboration and structural plasticity by NF-κB

**DOI:** 10.1016/j.tins.2011.03.001

**Published:** 2011-06

**Authors:** Humberto Gutierrez, Alun M. Davies

**Affiliations:** Cardiff School of Biosciences, University of Cardiff, Biomedical Sciences Building 3, Cardiff CF10 3AT, UK

## Abstract

The nuclear factor-kappa B (NF-κB) family of transcription factors has recently emerged as a major regulator of the growth and elaboration of neural processes. NF-κB signaling has been implicated in controlling axon initiation, elongation, guidance and branching and in regulating dendrite arbor size and complexity during development and dendritic spine density in the adult. NF-κB is activated by a variety of extracellular signals, and either promotes or inhibits growth depending on the phosphorylation status of the p65 NF-κB subunit. These novel roles for NF-κB, together with recent evidence implicating NF-κB in the regulation of neurogenesis in the embryo and adult, have important implications for neural development and for learning and memory in the mature nervous system.

## Introduction

The nuclear factor-kappa B (NF-κB) family of transcription factors is ubiquitously expressed, but it has been studied most extensively in the mammalian immune system where it plays a key role in regulating the expression of genes involved in innate and adaptive immune responses, inflammatory responses, cell survival and cell proliferation [1]. NF-κB proteins are widely expressed in the developing and mature nervous system, and studies of NF-κB reporter mice have revealed NF-κB activity in multiple brain regions at various stages of development and in the adult [2,3]. A wide variety of extracellular signals regulate NF-κB activity in the nervous system including neurotransmitters, neuropeptides, neurotrophins, cytokines and neural cell adhesion molecules (NCAMs) [2,4]. NF-κB has been implicated in regulating the expression of an increasing number of genes involved in neural development and/or function, for example, those encoding the NCAM [5] and receptors for some neurotransmitters, neuropeptides and neurotrophic factors [6–10]. NF-κB signaling is involved in several aspects of neural development including the regulation of neurogenesis in the embryo and adult [11–14], the regulation of cell survival in certain populations of peripheral and central neurons [2,15] and in promoting peripheral nerve myelination [16,17]. In the mature nervous system, a substantial body of evidence has implicated NF-κB signaling in diverse aspects of learning and memory [3,4]. Furthermore, inducible NF-κB participates in the cellular responses to neuronal insult and neurological diseases with beneficial or detrimental consequences. For example, in a variety of *in vitro* and *in vivo* models, NF-κB has been shown to have a neuroprotective function in apoptosis induced by glutamate, kainate, oxidative stress and amyloid β peptide [18]. Conversely, NF-κB has been implicated in enhancing neuronal apoptosis associated with ischemic brain injury, neurodegenerative diseases and inflammatory conditions [19].

In this review, we discuss emerging evidence for a major new role for NF-κB in regulating the growth and morphology of neural processes. After introducing the NF-κB family and the basics of NF-κB signaling, we provide an in-depth analysis of the ways in which NF-κB affects the elaboration and structural modification of neural processes. Furthermore, we discuss the broader significance of these findings and other recent advances with respect to the neurodevelopmental roles of NF-κB and its importance in learning and memory.

## The NF-κB family and NF-κB signaling pathways

In mammals, the NF-κB family of transcription factors consists of five structurally related proteins, p65, RelB, c-Rel, p50 and p52, that form homodimers and heterodimers that induce or repress gene expression by binding to DNA sequences (κB elements) within the promoters and enhancers of target genes [20]. NF-κB dimers are held in an inactive form in the cytosol by interaction with a member of the IκB family of proteins (see Box 1). NF-κB is activated by removal of the inhibitory IκB protein and translocation of the liberated NF-κB dimer to the nucleus. The predominant transcriptionally active form of NF-κB in the nervous system is the p65/p50 heterodimer whereas IκBα is the most common inhibitor.

**Figure 1:**
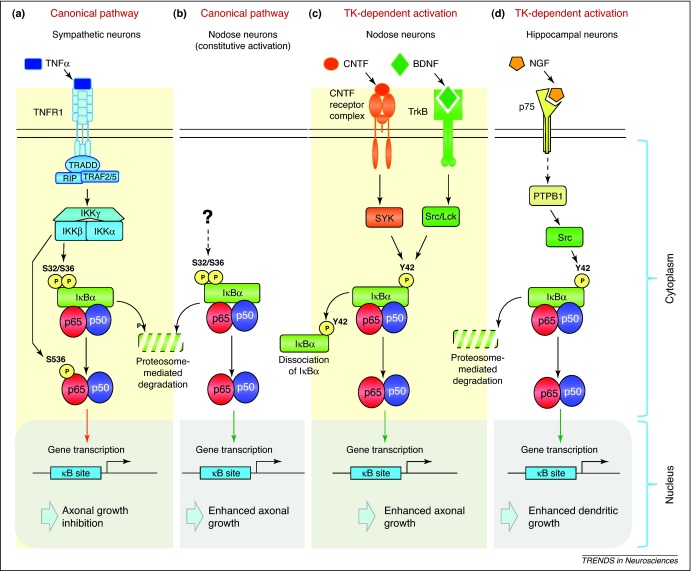
NF-κB signaling pathways regulating neurite growth from cultured neurons. In the canonical pathway **(a,b)**, activated IKKβ phosphorylates (P) IκBα at Ser32 and Ser36, leading to its ubiquitination and degradation by the proteasome, allowing the released p65/p50 dimer to translocate to the nucleus. IKKβ can also phosphorylate p65 at the Ser 536 residue, which switches NF-κB to a neurite growth-inhibitory function. Of the many extracellular signals that activate the canonical pathway, TNFα binding to tumor necrosis factor receptor-1 (TNFR1) is among the most extensively studied [1] and is known to operate in sympathetic neurons with resultant p65 phosphorylation and axonal growth inhibition (**a**) [37]. TNFR1 ligation results in receptor trimerization and the recruitment of several adapter proteins (TNFR1-associated death domain protein, TRADD; receptor interacting protein 1, RIP1; TNFR-associated factors, TRAF2 and TRAF5) and the IKK complex, which results in IKKβ phosphorylation by the participation of additional proteins recruited to the signaling complex (not shown). Canonical signaling without significant levels of phospho-S536–p65 facilitates BDNF-promoted axonal growth from nodose ganglion neurons during an E18–P1 developmental window (**b**) [33], although the upstream activators of this pathway have yet to be identified. In the TK-dependent pathway **(c,d)**, IκBα is phosphorylated at Tyr42, resulting in its dissociation (with or without degradation) from the p65/p50 dimer and translocation of the latter to the nucleus, and subsequent enhancement of neurite growth. In nodose ganglion sensory neurons **(c)**, binding of BDNF to TrkB during an E15 to E17 developmental window results in phosphorylation of IκBα by Src and Lrk [35], whereas CNTF signaling during perinatal development activates the tyrosine kinase SYK, which in turn phosphorylates IκBα, leading to its removal without proteasome-dependent degradation [34]. In hippocampal neurons **(d)**, binding of NGF to the p75^NTR^ receptor results in PTPB1-dependent phosphorylation of IκBα by the tyrosine kinase Src [53].

A great variety of stimuli activate NF-κB in different cell types via several distinct pathways (Figure 1). In the canonical NF-κB signaling pathway, NF-κB is activated by phosphorylation of IκBα on Ser32 and Ser36 by the IκB kinase-β (IKKβ) catalytic subunit of an IκB kinase (IKK) complex that consists of IKKβ together with another catalytic subunit, IKKα and a regulatory subunit IKKγ. This leads to ubiquitylation and proteasome-mediated degradation of IκBα and translocation of the liberated NF-κB dimer to the nucleus [20]. In the non-canonical NF-κB signaling pathway, the p100 subunit of p100–RelB heterodimers is phosphorylated by the IKKα subunit of the IKK complex, which results in ubiquitylation and proteosome-dependent processing of p100 to p52, followed by translocation of the resulting p52–RelB dimers to the nucleus. An additional IKK-independent NF-κB activation mechanism has been described in which IκBα is phosphorylated on Tyr 42, which results in dissociation of IκBα from NF-κB and translocation of NF-κB to the nucleus. We will refer to this NF-κB activation mechanism as tyrosine kinase-dependent (TK-dependent) NF-κB signaling.

The function of nuclear NF-κB is controlled by numerous post-translational changes to the NF-κB subunits, including phosphorylation and acetylation on specific residues that can affect transcriptional activity, target gene specificity or termination of the NF-κB response [21]. In addition to being modified and functionally modulated by other signaling pathways, components of the NF-κB signaling system affect the function of many other signaling proteins and transcription factor systems in the cell. IKKα and IKKβ, in particular, phosphorylate a wide variety of substrates in many other signaling pathways, and therefore have wide-ranging effects on cellular function [21].

## Regulation of the growth and morphology of neural processes

The growth, branching and spatial arrangement of axons and dendrites in the developing nervous system is regulated by a multitude of extracellular proteins and by changing patterns of neural activity as neurons establish and modify synaptic connections [22,23]. Extracellular signals and neural activity trigger changes in intracellular signaling networks that control the assembly and organization of the growth cone cytoskeleton and engage transcription factors that modulate the expression of genes required for growth. Extensive work over the past two decades has demonstrated the participation of several pivotal intracellular signaling proteins and transcription factors in the regulation of axon and dendrite growth, including phosphatidylinositol-3 kinase, glycogen synthase kinase 3, calcium/calmodulin-dependent protein kinases (CaMKs), mitogen activated kinases, cyclic AMP response element binding protein (CREB) and nuclear factors of activated T-cells [24].

Dendritic spines, the small protrusions that are the site of most of the excitatory synapses on principal neurons of the central nervous system (CNS), rapidly accumulate with the growth and elaboration of dendritic arbors [25]. These dynamic structures are turned over at a relatively high rate during development with a net gain in number into early postnatal development, after which substantial numbers are lost. Although spine number remains relatively constant during adulthood and the great majority of spines are long lasting and underpin long-term information storage [26], spine turnover continues at a low rate throughout life [25]. Dendritic spines also exhibit rapid, activity-dependent changes in size and shape [27]. For example, the dendritic spines of hippocampal pyramidal neurons increase in volume during long-term potentiation (LTP) and shrink during long-term depression (LTD) [28]. Although LTD can occur without obvious structural changes in spines [29], activity-dependent structural plasticity in dendritic spines is thought to contribute to changes in synaptic strength [27]. These findings together with the demonstration that changes in sensory experience can lead to persistent changes in spine density [28,30] and that instructive experiences lead to spine stabilization and enlargement [31,32] have strongly implicated structural spine plasticity in learning and memory.

In the following sections, we describe recent work that implicates NF-κB as a major player in the intracellular signaling and transcriptional networks that regulate the growth and elaboration of axons and dendrites during development (Table 1). This work, together with recent reports implicating NF-κB signaling in the regulation of dendritic spine density, are subsequently discussed in the context of the broader significance of NF-κB in learning and memory.

## NF-κB in the regulation of axon growth and branching

**Figure 2:**
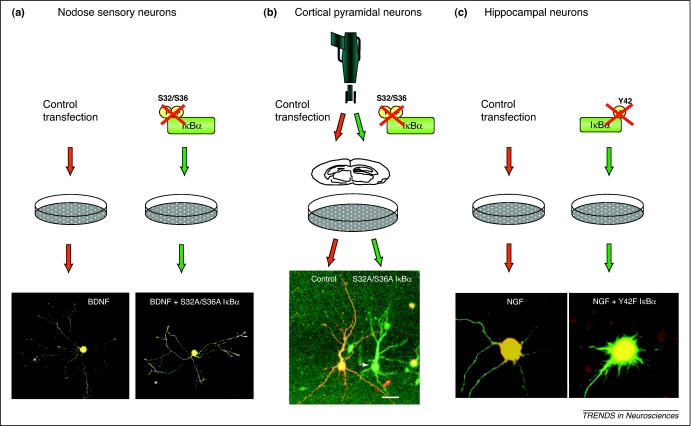
Effects of selectively inhibiting NF-κB signaling pathways on axon and dendrite growth and morphology. Examples of selectively inhibiting the canonical signaling pathway in dissociated cultures of **(a)** dissociated nodose ganglion neurons cultured with BDNF and **(b)** pyramidal neurons in somatosensory cortical slice cultures by ballistic transfection with a plasmid expressing a super-repressor S32A/S36A IκBα. In the slice cultures, S32A/S36A IκBα-transfected and control-transfected neurons were identified in the same preparation by co-transfection with plasmids expressing green and red florescent proteins, respectively. Inhibition of NF-κB canonical signaling significantly reduced the size and complexity of the respective axon and dendrite arbors of these sensory and pyramidal neurons. **(c)** Examples of dissociated hippocampal neurons cultured with NGF and transfected with control and Y42F–IκBα-expressing plasmids to selectively inhibit the TK-dependent activation pathway. Inhibition of this pathway resulted in an increase in the number of primary dendrites and a reduction in dendrite elongation. Reproduced, with permission, from [33] (panels **a** and **b**) and [54] (panel **c**).

Evidence for the regulation of axonal growth by NF-κB has come from studying the effects of manipulating NF-κB signaling pathways in primary cultures of nodose ganglion sensory neurons established from developing mice (Figure 2a). These neurons survive and extend long branching axons on laminin-coated culture dishes in medium containing either brain-derived neurotrophic factor (BDNF) or ciliary neurotrophic factor (CNTF). From a series of detailed developmental studies of these neurons [33–36], a complex picture has emerged in which the NF-κB signaling pathway affecting axon growth and its mechanism of activation depends on the developmental stage of these neurons and the neurotrophic factor used to support their survival.

Although NF-κB signaling makes a major contribution to BDNF-promoted neurite growth throughout late fetal and early postnatal development, shortly before birth there is an abrupt switch in the NF-κB signaling network required for BDNF-promoted neurite growth. During a narrow developmental window between embryonic day 15 (E15) and E17, BDNF strongly activates the TK-dependent NF-κB signaling pathway through activation of its receptor TrkB and subsequent activation of two members of the Src family of tyrosine kinases, Src and Lck. These kinases in turn phosphorylate IκBα on Tyr 42 [35] (Figure 1c). Blocking the TK-dependent NF-κB signaling pathway, but not the canonical signaling pathway, during this window of development significantly reduces BDNF-promoted axon growth and branching [35].

Subsequently, during a narrow developmental window between E18 and postnatal day 1 (P1), blocking the canonical signaling pathway, but not the TK-dependent NF-κB signaling pathway, significantly reduces BDNF-promoted axon growth and branching [33]. BDNF does not activate NF-κB signaling during this latter period of development, and the mechanism by which NF-κB transcriptional activity is maintained in nodose neurons during this period is not yet understood [33] (Figure 1b). The relatively high *in vivo* levels of phospho-Tyr42-IκBα in late fetal nodose ganglia and the subsequent marked decrease in the level of IκBα phosphorylated at Tyr42 residue during the early postnatal period is consistent with the *in vivo* operation of the TK-dependent NF-κB activation mechanism prenatally and its subsequent loss postnatally [35]. The reason for the switch and developmental plasticity in the NF-κB signaling network required for BDNF-promoted axon growth is unclear, but it may be necessary to facilitate other physiological or developmental changes occurring in the neurons over this period. Interestingly, tumor necrosis factor superfamily member 14 (TNFSF14/LIGHT) acting via its receptor TNFRSF14/HVEM is a potent inhibitor of NF-κB signaling and negative regulator of BDNF-promoted neurite growth from nodose neurons during the E18–P1 developmental window [36].

Like BDNF, CNTF also activates the TK-dependent NF-κB signaling pathway in developing nodose neurons [34], but unlike BDNF, which activates NF-κB only during a narrow window of late fetal development, CNTF potently activates NF-κB in both fetal and postnatal neurons [35]. CNTF treatment activates spleen tyrosine kinase (SYK), which in turn phosphorylates IκBα on Tyr 42, leading to increased NF-κB transcriptional activity. Blocking either SYK activation or the TK-dependent NF-κB signaling pathway, but not the canonical signaling pathway, virtually eliminates CNTF-promoted axonal growth, demonstrating a crucial requirement for NF-κB signaling in axonal growth in response to this neurotrophic factor [34] (Figure 1c). Taken together, the above studies on developing sensory neurons indicate that different extracellular regulators of axon growth converge on the NF-κB signaling network in various ways at different stages in development to enhance axon growth and branching.

In addition to enhancing growth, NF-κB signaling can also exert a potent inhibitory effect on axon growth. Whether NF-κB promotes or inhibits growth depends on the mechanism of NF-κB activation and the resulting phosphorylation status of the p65 NF-κB subunit. Neonatal sympathetic neurons, which survive and extend axons in response to the neurotrophin nerve growth factor (NGF), contain p65 that is constitutively phosphorylated on Ser 536 (phospho-S536–p65). Inhibiting NF-κB transcriptional activity in these neurons does not affect NGF-promoted neurite growth, and enhancing NF-κB transcriptional activity by tumor necrosis factor α (TNFα) treatment or by overexpressing p65, p65/p50, or IKKβ strongly inhibits NGF-promoted neurite growth [37] (Figure 1a). By contrast, neonatal nodose neurons contain barely detectable levels of phospho-S536-p65 protein, and inhibiting NF-κB transcriptional activity in these neurons reduces BDNF-promoted neurite growth, whereas enhancing NF-κB transcriptional activity by overexpressing p65/p50 increases neurite growth [37]. However, enhancing NF-κB transcriptional activity in nodose neurons by overexpressing IKKβ, which additionally phosphorylates p65 on Ser 536, strongly inhibits BDNF-promoted neurite growth [37]. The demonstration that an S536D–p65 phosphomimetic mutant also inhibits neurite growth in nodose neurons and that blockade of Ser 536 phosphorylation by a S536A–p65 mutant protein prevents the growth inhibitory effects of IKKβ overexpression in both nodose and sympathetic neurons indicate that the phosphorylation of p65 on Ser 536 is a key determinant of whether NF-κB signaling promotes or inhibits neurite growth [37]. Interestingly, BDNF treatment of fetal nodose neurons between E15 and E17 not only activates TK-dependent NF-κB signaling but also promotes dephosphorylation of p65 on S536, both of which facilitate axon extension [35]. Although the molecular mechanism by which the phosphorylation status of p65 affects axon growth is unknown, it seems likely that phosphorylated S536–p65 and non-phosphorylated S536–p65 regulate different sets of genes that have opposing effects on axon growth. Ser 536 is located within a C-terminal transactivation domain of p65, and there is evidence that the phosphorylation status of S536 in p65 differentially affects which sets of genes will be transcribed [38].

Although inhibiting NF-κB activity in neonatal sympathetic neurons does not affect NGF-promoted neurite growth [37], in the PC12 pheochromocytoma cell line, which responds to NGF by exiting the cell cycle and differentiating into sympathetic neuron-like cells, inhibiting NF-κB signaling impairs differentiation into cells with short neurite-like processes [39–41]. NGF activates NF-κB signaling in PC12 cells [42], and enhancing NF-κB signaling in these cells in the absence of NGF by overexpressing NF-κB inducing kinase, an upstream activator of IKKα, or by expression of a constitutively active form of IKKβ increases the percentage of neurite-bearing cells [43,44]. In contrast to PC12 cells, normal sympathetic neuroblasts exit the cell cycle and differentiate into neurons independently of NGF and only become responsive to this neurotrophin after their axons reach their targets *in vivo* [45]. Nonetheless, these intriguing findings in PC12 cells raise the possibility that NF-κB signaling may play some role in regulating the differentiation of sympathetic neuroblasts and/or promoting the growth of neurites from neuroblasts and early sympathetic neurons. For these reasons, it will be informative to explore the effects of manipulating NF-κB signaling on proliferation, differentiation and *de novo* neurite growth in primary cultures of sympathetic neuroblasts and early post-mitotic sympathetic neurons.

In addition to regulating the growth of sensory and sympathetic axons during the stage of development when they are growing and branching extensively under the influence of target-derived neurotrophins, NF-κB signaling has recently been implicated in the earlier stages of axon initiation and guidance. In cultures of E17 mouse hippocampal neurons, phospho-S32/36–IκBα is selectively distributed along the length of early axons and becomes progressively concentrated in the axon initial segment (AIS) with axon elongation [46]. Phospho-IKKβ is present in these early neurons, and pharmacological inhibition of IKK significantly reduces the level of phospho-IκBα and significantly impairs axon formation without affecting the development of minor neurites [46]. Phospho-IκBα immunoreactivity in the AIS of hippocampal and cortical neurons increases from prenatal to postnatal stages [46]. Furthermore, phospho-IκBα, activated IKK and phospho-S536–p65 are expressed in the AIS of a wide variety of neurons in other adult rat CNS regions [47], raising the possibility that NF-κB signaling might play a role in axogenesis of many different neuronal subtypes. Whether activated IKK and/or phospho-IκBα promote AIS specification and/or early axon extension in developing hippocampal neurons by a local effect on the cytoskeleton and/or by retrograde NF-κB signaling to the nucleus with changes in gene expression is unclear. IKK, for example, regulates F-actin assembly [48] and β-catenin stability [49], and β-catenin has been implicated in axon initiation [50]. Evidence for a role for NF-κB signaling in axon guidance has come from the observation that misexpression of Dorsal, an NF-κB protein in *Drosophila*, causes photoreceptor axons to reach incorrect positions within the optic lobe [51].

## NF-κB in the regulation of dendritic growth and morphology

Several studies have demonstrated a role for NF-κB signaling in regulating the growth and morphology of dendritic arbors in several regions of the developing and mature CNS. In the intact neuropil of somatosensory cortical slice cultures prepared from P3/P4 mice, inhibiting either NF-κB canonical signaling or NF-κB-mediated transcriptional activation causes highly significant reductions in dendritic length and branching of layer 2/3 pyramidal neurons [33] (Figure 2b). Pharmacological inhibition of IKK in E17 dissociated rat hippocampal neurons also significantly reduces dendritic length and branching [52]. A particularly thorough and detailed series of studies in E17 dissociated mouse hippocampal neurons has revealed a chain of molecular events linking neurotrophin stimulation to changes in dendrite growth via sequential activation of NF-κB and other transcription factors. In low-density cultures, NGF reduces the number of primary dendrites and promotes dendrite elongation by binding to the common neurotrophin receptor (p75^NTR^) [53] (Figure 2c). This leads, by an unknown mechanism, to the activation of protein tyrosine phosphatase 1B, which activates protein tyrosine kinases, including Src, that phosphorylate Tyr42 of IκBα, resulting in NF-κB activation [54] (Figure 1d). Selectively inhibiting TK-dependent NF-κB signaling, but not canonical signaling, inhibits the effects of NGF on dendrite morphology in these cultures.

NF-κB-dependent enhanced expression of the helix-loop-helix transcription factors, Hes1 and Hes5, plays a key role in bringing about the effects of NGF on dendrite number and growth [53]. These effects of NGF on dendrites are also partly dependent on NGF-promoted decrease in the expression of the proneural basic helix-loop-helix transcription factors, neurogenin-3 and Mash1 [55]. Because Hes proteins are repressors of proneural gene transcription [56], it is possible that NF-κB activation initiates a transcription factor cascade that mediates the effects of neurotrophins on dendrite morphology. Interestingly, Notch signaling, which is also a key regulator of dendrite growth, also regulates Hes1/5 expression. Indeed, Notch and two of its ligands, Delta-1 and Jagged-1, are expressed together with p75^NTR^ and Hes1/5 in CA3 pyramidal neurons of the hippocampus [55], suggesting convergence of Notch and neurotrophin-regulated NF-κB signaling in the control of dendritic morphology, at least in this brain region.

Evidence for the participation of NF-κB in dendritogenesis in the developing cerebellum has come from analysis of the *Purkinje cell degeneration* mutant mouse, *psd*^*Sid*^, which has an exon 7 deletion in *Nna1* and is characterized by underdevelopment of Purkinje cell dendrites [57]. The Purkinje cells of these mice have reduced expression and nuclear localization of p65 both *in vivo* and *in vitro*. Knockdown of p65 by short hairpin RNA (shRNA) in developing wild type Purkinje cells in culture mimics the detrimental effect of Nna1 knockdown on dendritic arbor size and complexity [57]. Moreover, p65 overexpression was observed to completely reverse the effects of Nna1 knockdown on dendrite growth [57].

It has recently been reported that manipulating the function of IKKβ in medium spiny neurons (MSNs) of the nucleus accumbens (NAc) of adult mice in the context of reward and stress behavioral paradigms influences dendritic spine density and morphology [58,59]. Viral-mediated transfection of MSNs with a constitutively active IKKβ (caIKKβ) mutant that activates NF-κB signaling was found to increase the number of dendritic spines in these neurons whereas a dominant-negative IKKβ (dnIKKβ) mutant decreased spine number [58]. Chronic cocaine exposure also increased spine number and induced NF-κB-dependent transcription in the NAc. The demonstration that dnIKKβ blocked both the ability of cocaine to increase spine number and its rewarding effects suggests that IKK participates in regulating the structural and behavioral plasticity that accompanies chronic cocaine exposure [58]. Chronic social defeat stress has been shown to increase the levels of IKK, IκB and phospho-IκB proteins in the NAc and to increase the density of stubby-type spines in the MSNs of a subset of animals that respond to such stress with social avoidance [59]. The finding that the dnIKKβ viral vector reverses both the increased formation of stubby spines and social avoidance in susceptible animals and that the caIKKβ viral vector promotes social avoidance in mice subjected to a subthreshold social defeat regime suggests that IKK plays a role in regulating stress-induced adaptive plasticity in the NAc [59]. It should be noted, however, although bicistronic herpes simplex viral vectors were used to manipulate IKK function and identify IKK-transfected MSNs in both studies, because these vectors infect a variety of cell types, it is uncertain whether the observed changes in spine density and morphology resulted exclusively from interfering with IKK function in MSNs themselves. Furthermore, since IKK function affects a variety of signaling pathways in the cell in addition to modulating NF-κB activation [20], because NF-κB transcriptional activation was not specifically blocked in these studies, it remains to be determined to what extent the observed changes in dendritic spine density and morphology were mediated by NF-κB-regulated changes in gene expression.

## NF-κB transcriptional targets in the regulation of axonal and dendritic growth

Although the available evidence suggests that the effects of NF-κB signaling on the growth of neural processes are brought about by NF-κB-dependent changes in gene transcription, the relevant transcriptional targets are largely obscure. Only in the case of Purkinje dendritic growth have two of the required NF-κB transcriptional targets been identified, namely those encoding the microtubule-associated proteins, MAP2 and MAP1B [57]. These proteins are mainly localized in the somatodendritic compartment of neurons where they promote microtubule assembly and stabilization, and have been shown to play a key role in dendrite elongation in developing hippocampal neurons [60,61]. The underdeveloped Purkinje dendrites of *psd*^*Sid*^ mice exhibit reduced expression of MAP2 and MAP1B, and shRNA knockdown of these proteins in wild type cerebellar neurons also suppresses dendrite growth in culture [57]. Knockdown of p65 in wild type cerebellar neurons results in reduced expression of MAP2 and MAP1B, and the detrimental effect of p65 knockdown on dendrite growth is prevented by overexpressing MAP2 and MAP1B [57]. Although these findings suggest that MAP2 and MAP1B play a role in mediating the effects of NF-κB signaling on dendrite growth, they do not reveal if additional NF-κB-regulated genes are required. Furthermore, these dendritically-localized proteins are unlikely to have any bearing on how NF-κB affects axonal growth.

Of the multitude of genes regulated by NF-κB in various cell types, several encode proteins that have been implicated in cell adhesion and neurite growth, such as β1 integrin [62], NCAM [5], slit and Trk-like family member 1 and T-lymphoma and metastasis 1 [52]. NF-κB also regulates the expression of glial cell-derived neurotrophic factor (GDNF) family receptor α1, which influences the responsiveness of Neuro2a neuroblastoma cells to GDNF [9]. In addition to evaluating the importance of particular NF-κB transcriptional targets in mediating the effects of NF-κB signaling on axon and dendrite growth, transcriptome profiling of neurons under experimental conditions in which NF-κB signaling either promotes or inhibits growth is likely to provide additional NF-κB transcriptional targets that are candidates for mediating NF-κB-regulated structural plasticity.

## Implications for learning and memory of the effects of NF-κB on structural plasticity, neurogenesis and gene transcription

Numerous pharmacological and genetic studies utilizing a variety of experimental paradigms have implicated NF-κB signaling in learning and memory in diverse species. For example, pharmacological inhibition of NF-κB interferes with memory formation in the crab [63], and administration of NF-κB decoy DNA interferes with long-term fear memory [64], long-term inhibitory avoidance memory [65] and long-term spatial memory [66] in rodents. Genetic evidence for the participation of NF-κB signaling in learning and memory has come from the analysis of several knockout, conditional knockout and conditional overexpression mouse lines (for a review, see [3]). For example, *p50*^−/−^ mice have a selective deficit in short-term spatial memory performance [13], *c-rel*^−/−^ mice have impaired hippocampus-dependent memory formation [67], and defects in spatial learning have been observed in *p65*^–/–^/*Tnfr1*^−/−^ mice [68] and CamKII-tTAtetOtn IκBα mice, which conditionally express the super-repressor IκBα in forebrain glutamatergic neurons [69]. Conversely, Prion-tTAtetOtn IκBα mice that conditionally express high levels of the super-repressor IκBα in GABAergic neurons, and at lower levels in glutamatergic neurons, display increased exploratory activity and enhanced spatial learning and memory [70]. Whereas reduced LTP and LTD are evident in CamKII-tTAtetOtn IκBα mice [69] and late-phase LTP is impaired in *c-rel*^−/−^ mice [67], enhanced LTP is observed in Prion-tTAtetOtn IκBα mice [69].

Links between synaptic function and NF-κB signaling that are potentially relevant for learning and memory have been increasingly recognized. The p65 and p50 NF-κB subunits are detectable at pre- and postsynaptic sites *in vivo* and in synaptosomes isolated from the cerebral cortex and hippocampus [68,71]. Glutamatergic stimulation of cultured cerebellar granule cells and hippocampal neurons, or isolated hippocampal synaptosomes, enhances NF-κB DNA binding activity, whereas pharmacological inhibition of ionotropic glutamate receptors or L-type Ca^2+^ channels suppresses basal NF-κB activity [68,72,73]. NF-κB is also activated in the mouse hippocampus by perforant path LTP *in vivo* [74]. Following glutamatergic stimulation of cultured hippocampal neurons, NF-κB is conveyed from distal dendrites to the nucleus by active, dynein-dependent transport [66,75–77]. Collectively, these studies indicate that NF-κB is capable of transducing changes in synaptic activity to transcriptional changes in the nucleus. One NF-κB target gene that may provide a potentially relevant link with learning and memory is the α catalytic subunit of protein kinase A (PKA) [69]. PKA and one of its substrates, CREB, play key roles in the formation of long-lasting memories [78]. Although PKA expression is reduced and forskolin-induced CREB phosphorylation is impaired in the hippocampus of CamKII-tTAtetOtn IκBα mice [69], alternative signaling pathways link synaptic activity and various extracellular signals to CREB activation [78]. Therefore, although NF-κB and CREB participate in a transcriptional network involved in aspects of learning and memory, they do not necessarily act in a mutually dependent sequential manner.

Alternative ways in which NF-κB signaling could affect learning and memory relate to its roles in regulating the growth and morphology of axons and dendrites, as discussed above. In particular, the possible influence of NF-κB signaling on dendritic spine number and morphology in the adult NAc *in vivo* [58,59] is pertinent given the relationship between LTP/LTD induction and structural plasticity of dendritic spines [79]. For these reasons, it will be important to examine whether NF-κB signaling is capable of influencing spine turnover and morphology in other neurons in the developing and mature brain, and to ascertain in appropriate mouse genetic and other models if NF-κB signaling in the hippocampus contributes to structural plasticity of dendritic spines associated with learning and memory. Moreover, the recognition that NF-κB affects the elaboration of axons and dendrites during development has potential implications for the interpretation of studies of learning and memory in certain mouse genetic models that lack key components of the NF-κB signaling system from conception. The potential consequences of defective NF-κB during development may influence the performance on a particular learning and memory task in the adult in ways that are unrelated to the normal functions of NF-κB signaling during training and recall in wild type mice.

An additional way in which NF-κB signaling might influence learning and memory relates to its role in neurogenesis (Table 1). Several recent studies have implicated NF-κB signaling in regulating neurogenesis during development and in the adult. NF-κB mediates the neurogenic effect of erythropoietin in neurosphere cultures of neural stem cells (NSCs) established from E14 mouse ganglionic eminence [80]. Additionally, cell proliferation is impaired in neurosphere cultures established from E13 mouse embryos that lack p65 and p50, compared with cultures established from wild type mice [11].

In the adult CNS, neurogenesis occurs in the subventricular zone (SVZ) of the lateral wall of the lateral ventricle and in the subgranular zone (SGZ) of the dentate gyrus. In adult rats, stress activates interleukin-1β (IL-1β)/IL-1RI signaling, followed by the activation of NF-κB in NSCs in the SGV [14]. Inhibiting either IL-1β/IL-1RI signaling or NF-κB activation prevents stress-induced suppression of hippocampal NSC proliferation, but does not affect NSC proliferation in non-stressed rats [14,81]. Although NSC proliferation is likewise unaffected in the SGV of adult *p50*^–/–^ mice, there is a marked deficiency in neurogenesis in the hippocampus of these mice as a result of a defect in the late maturation of newly born neurons [13]. Inhibition of NF-κB in cultured adult neural progenitor cells also significantly impairs their differentiation and inhibits the differentiation of these cells by toll-like receptor 4 (TLR4) activation, a known activator of NF-κB [82]. NF-κB also mediates the *in vitro* proliferative response of adult SVZ NSCs to TNFα [12] and activation of the multi-ligand receptor RAGE (receptor for advanced glycation endproducts) [83], which may be relevant for enhancing NSC proliferation in brain inflammation and injury [84].

Numerous studies demonstrating increased hippocampal neurogenesis following hippocampus-dependent, but not hippocampus-independent, learning tasks together with the detrimental effects of neurogenesis suppression on performance of certain hippocampus-dependent learning tasks, has established a potential link between neurogenesis in the adult hippocampus and learning and memory [85]. The deficiency in hippocampal neurogenesis and selective defect in short-term spatial memory performance in adult *p50*^–/–^ mice [13] together with evidence for the preferential recruitment of adult-generated granule cells into spatial memory networks in the dentate gyrus [86] is consistent with, but by no means proves, a contribution of NF-κB-regulated hippocampal neurogenesis to an aspect of hippocampal-dependent learning. Studies of adult hippocampal neurogenesis in mice that have conditional mutations in various components of the NF-κB signaling pathway combined with behavioral studies of hippocampal-dependent learning tasks, may shed further light on the potential link between NF-κB-regulated neurogenesis and learning and memory.

## Conclusions

NF-κB signaling is becoming increasingly recognized as a major regulator of the growth and morphology of neural processes in the developing and mature nervous system. From the small number of peripheral and central neurons that have so far been investigated, NF-κB has been implicated in axonal and dendritic growth regulation from the earliest stages of process initiation to stages when neurons are establishing functional connections during development to the regulation of dendrite spine number in adult neurons. These observations raise many outstanding questions relating to the regulation and modulation of NF-κB signaling and the identification of relevant NF-κB target genes that influence the development and structural plasticity of neural processes (Box 2). To date, most of the work on the influence of NF-κB signaling on the growth and elaboration of neural processes has been carried out in cultured primary neurons, and it will be important to clarify the physiological relevance of these observations *in vivo* using viral vectors to manipulate NF-κB signaling and by using conditional gain-of-function or loss-of-function approaches in transgenic mice. Such studies will likely reveal important insights into the transcriptional networks that regulate the establishment and refinement of neuronal morphology and neural circuitry during development and structural plasticity in the mature nervous system.

## Figures and Tables

**Figure I:**
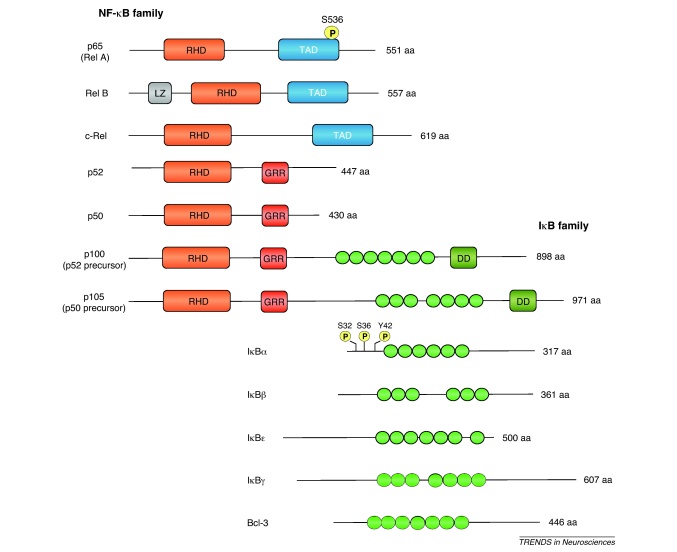
Schematic diagram illustrating the mammalian members of the NF-κB and IκB families.

**Table 1:** Influence of NF-κB signaling on the generation and morphological differentiation of neurons

NF-κB-regulated cellular function	Cell type and stage of development	Refs
Neurogenesis	• Embryonic NSCs	[11,80]
	• Adult NSCs	[12–14,82,83]
Cell differentiation	• Differentiation of proliferating PC12 cells into neuron-like cells	[39–41,43,44]
Axon initiation	• Embryonic hippocampal neurons	[46]
Axon guidance	• *Drosophila* photoreceptor axons	[51]
Axon growth and branching	• Perinatal nodose ganglion sensory neurons	[33–36]
Inhibition of axon growth and branching	• TNFα inhibition of NGF-promoted axon growth from perinatal sympathetic neurons	[37]
Dendritic growth and branching	• Postnatal cortical pyramidal neurons	[33]
	• Embryonic hippocampal neurons	[52–55]
	• Postnatal cerebellar Purkinje cells	[57]
Dendritic spine number and morphology	• Adult NAc medium spiny neurons	[58,59]

## Box 1 NF-κB and IκB families

The members of the mammalian NF-κB family, p65 (also known as RelA), RelB, c-Rel, p50 and p52, together with the p100 and p105 precursors of p52 and p50, share a conserved, multifunctional Rel homology domain (RHD) of approximately 300 amino acids that mediates dimerization, IκB protein binding and DNA binding (Figure I). The RHD contains a nuclear localization sequence that is masked by bound IκB protein and promotes nuclear translocation following IκB removal. p65, RelB and c-Rel additionally possess unrelated C-terminal transactivational domains (TAD), RelB has a leucine zipper (LZ), p52, p50 and their precursors have glycine-rich regions (GRR) and the latter precursors additionally possess regions with homology to death domains (DD). Phosphorylation of p65 at S536 switches activated NF-κB from a promoter to an inhibitor of neurite growth.

IκB proteins include p100, p105, IκBα, IκBβ, IκBɛ, IκBγ and Bcl-3. They contain several ankyrin-repeat motifs (denoted by green ovals) that mediate binding to RHDs. The ankyrin-repeat motifs in the C-terminal regions of the p105 and p100 act as auto-inhibitory IκB-like domains, retaining these precursors and their partner NF-κB proteins in the cytoplasm. Various phosphorylation sites (S32, S36 and Y42) within IκBα have been demonstrated to be important in regulating NF-κB activation. In contrast to the other IκB proteins, B-cell lymphoma-3 (BCL-3) is a nuclear protein that functions as a transcriptional co-activator.

## Box 2 Outstanding questions

• What is the physiological importance of the regulation of axonal and dendritic growth by NF-κB signaling for neuronal development *in vivo*, and is it relevant for only a subset of neurons or does it exert a ubiquitous influence?
• To what extent does NF-κB signaling affect dendritic spine structural plasticity in the adult nervous system and does it exert any effects on presynaptic terminals?
• To what extent do NF-κB-dependent changes in axonal and dendritic plasticity contribute to learning and memory?
• What are the *in vivo* physiological regulators of NF-κB signaling in neurons that are relevant for controlling the growth, elaboration and modification of neural processes?
• Which NF-κB-mediated changes in gene transcription are necessary and sufficient for promoting or inhibiting the growth of neural processes?
• Does NF-κB signaling affect the growth of axons and dendrites in distinctive ways in the same neurons? Can NF-κB negatively regulate dendritic growth in certain circumstances, as has been demonstrated for axons?
• In addition to phosphorylation of p65 at S536, do other post-translational modifications to NF-κB proteins influence the effects of NF-κB signaling on the growth and morphology of neural processes?
• In addition to transcriptional changes brought about by retrogradely transported activated NF-κB, does engagement of the NF-κB signaling network exert any local effects on the cytoskeleton that affect the morphology of axons and dendrites?
